# Structural studies and selected physical investigations of LiCoO_2_ obtained by combustion synthesis

**DOI:** 10.3762/bjnano.13.121

**Published:** 2022-12-07

**Authors:** Monika Michalska, Paweł Ławniczak, Tomasz Strachowski, Adam Ostrowski, Waldemar Bednarski

**Affiliations:** 1 Department of Chemistry and Physico-Chemical Processes, Faculty of Materials Science and Technology, VŠB-Technical University of Ostrava, 17. listopadu 15/2172, 708 00 Ostrava-Poruba, Czech Republic; 2 Łukasiewicz Research Network - Institute of Microelectronics and Photonics, Al. Lotników 32/46, 02-668 Warsaw, Poland; 3 Institute of Molecular Physics Polish Academy of Sciences, Smoluchowskiego 17, 60-179 Poznań, Poland

**Keywords:** lithium cobalt oxide, lithium-ion battery, nanocrystalline powder, solution combustion synthesis

## Abstract

Nanocrystalline powders of LiCoO_2_ were synthesized using a modified solution combustion method, and the effects of the annealing temperature (450–900 °C) on structure and composition were investigated using various methods, including XRD, SEM, EPR, and electrical studies. It was found that, as the process temperature increases, the value of the specific surface area decreases, and, hence, the size of the crystallites increases. XRD analysis showed that phase-pure LiCoO_2_ material was maintained without additional phases. EPR studies revealed the presence of two Ni^3+^ complexes resulting from Ni impurities. The electrical properties of the studied LiCoO_2_ samples were investigated by using impedance spectroscopy. Comparison of the effect of annealing temperature on electrical conductivity shows a very interesting behavior. As the annealing temperature increases, the DC conductivity value increases, reaching a maximum at a temperature of 500 °C. However, further increase in the annealing temperature causes a steady decrease in the DC conductivity.

## Introduction

Lithium cobalt oxide (LiCoO_2_, LCO) of hexagonal structure (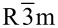) was first used as cathode material in lithium cells in 1979 by researchers from Oxford University [1]. The cell consisted of LCO, which was used as the cathode material, and metallic lithium, which was used as the anode material. In 1985, it was proposed to replace the Li metal in the negative electrode with the carbonaceous material graphite capable of reversibly intercalating lithium ions [2]. The commercialization of lithium-ion cells was achieved in the early 1990s by Sony Corporation and in 1992 by a joint venture company (Asahi Kasai and Toshiba) [2–4]. Almost 90% of commercial Li-ion batteries consist of a lithium cobalt oxide cathode and a graphite anode immersed in a lithium-ion conducting electrolyte, which is 1 M lithium hexafluorophosphate LiPF_6_ in a 1:1 (v/v) mixture of ethylene and dimethyl carbonate. Most commercial Li-ion cells are used to power portable devices, including mobile phones, laptops, and cameras [5–7].

One of the main advantages of the cobalt-based battery is its high theoretical capacity of 274 mAh·g^−1^, the high working potential of 4.0 V vs Li/Li^+^, and the high energy density of approximately 500 Wh·kg^−1^ [5–9]. The complete removal of lithium ions from the LiCoO_2_ structure is prevented by the phase transition from a hexagonal structure to a monoclinic structure, which occurs during cathode charging at a potential of approximately 4.2 V [5–9]. A decrease in capacity (approx. 50%) is observed during the cycling charging–discharging processes, caused by the dissolution of cobalt ions in the electrolyte above 4.2 V. Therefore, the practical capacity of the LCO material is approximately 150 mAh·g^−1^ [5–9]. One of the ways to improve the performance is (i) to obtain a nanosized LiCoO_2_ material in different forms and shapes using chemical or physical syntheses (see discussion below), (ii) the substitution of Co ions with other metal ions, such as Mg, Al, Fe, Ni, Mn, V [10–22], or (iii) the surface modification by carbon, metal, or oxide coatings [15–16].

Nanomaterials are preferred for the use in energy storage and conversion devices, such as Li-ion batteries, solar cells, solid oxide fuel cells, and thermoelectrics. Unusual and unexpected properties and also unique microstructures (and shapes), such as high porosity, high surface area, short reaction pathways, and diffusion length for Li-ion transport, eventually improve electrical conductivity and electrochemical performance [5–7,9,13–16,23–32]. Nanostructured materials can reduce the specific surface current rate as well as improve stability and specific capacity [23–29].

LiCoO_2_ has been produced in the form of powders, fibers, and films by using various processing techniques including wet chemical synthesis, such as the sol–gel method [33–35], precipitation [36], hydrothermal [37–39] and spray pyrolysis [40–41]. Also, solid-state synthesis methods, such as mechanical synthesis [42], thermal decomposition [43], or microwave synthesis [44–47] have been used.

Wet chemical synthesis allows for molecular-level mixing of the starting components, resulting in a very homogeneous product comprising fine particles and a large surface area. The stoichiometry of the product created by using wet techniques may be controlled with a greater precision than that of a product from a solid-state method. Combustion synthesis (CS), also known as self-propagating high-temperature synthesis (SHS), is a low-cost process for handling a wide variety of industrially relevant materials. CS is a widely used method for the creation of nanomaterials [48–57]. Acetates, carbonates, and nitrate salts of lithium and cobalt are often utilized as starting materials and oxidizers in the combustion synthesis of lithium cobalt oxide [50,58–59]. Different ammonium carboxylates were investigated as fuels, including ammonium acetate, ammonium citrate, or ammonium tartarate [55], urea [56], starch [57], citric acid [58], and 1,2-diformylhydrazine [59]. The obtained LCO precursors were annealed in air between 300 and 850 °C [55–59].

Herein, we demonstrate a new combustion solution synthesis (CSS) to obtain a single-phase nanocrystalline lithium cobalt oxide (LiCoO_2_, LCO) with layered structure. We investigated the relationships between the heating temperature and (i) structural parameters (crystallite sizes, lattice parameters, and volume cells) by using XRD analysis, (ii) morphology (size and distribution of grains) by using SEM, (iii) specific surface area (SSA) by carrying out Brunauer–Emmett–Teller (BET) measurements, (iv) oxidation states of metals by measuring electron paramagnetic resonance (EPR), and (v) electrical parameters (thermal dependencies of conductivity and comparison of electrical properties of LCO powders) by using impedance spectroscopy (IS). Our studies provide important information on the mechanism of formation, particle growth, size, shape, and the control of these parameters during synthesis.

## Materials and Methods

### Synthesis of LiCoO_2_ powders

25.408 g of high-purity cobalt(II) acetate tetrahydrate (C_4_H_6_O_4_Co·4H_2_O, reagent grade), 10.408 g of lithium acetate dihydrate (C_2_H_3_O_2_Li·2H_2_O, purum p.a., crystallized, 97.0% (NT)) and 20 g of ᴅ-(+)-glucose (C_6_H_12_O_6_, ≥99.5% (GC), Sigma-Aldrich) were used to synthesize LiCoO_2_ nanocrystalline powders.

**Figure 1:**
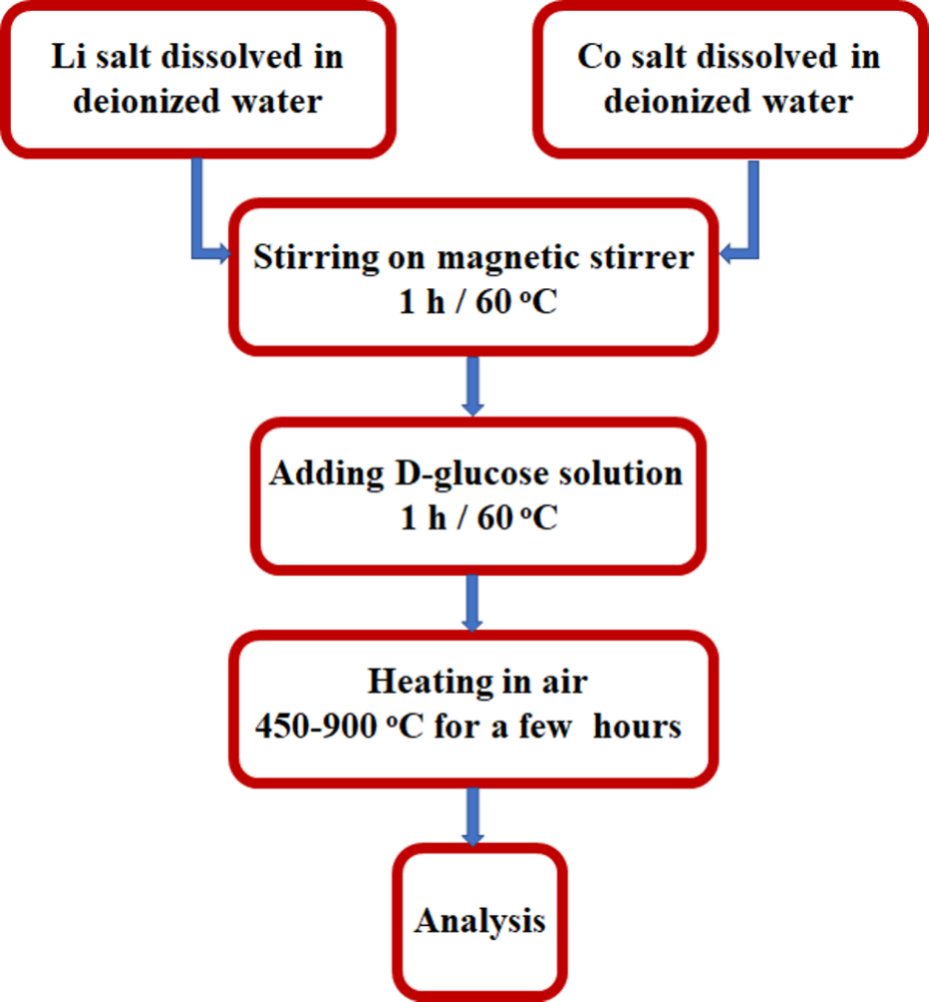
Flowchart for combustion solution synthesis (CSS) of LiCoO_2_.

Cobalt and lithium acetate salts were dissolved separately in small amounts of deionized water. Then, the solutions were mixed together and a solution of ᴅ-(+)-glucose was added. The prepared solutions were then evaporated until a gel was obtained. The resulting gel precursor was heated from 450 to 900 °C for 5 h in air. The heating rate was 5 °C/min. The synthesis flowchart is presented in Figure 1.

### Characterization of LiCoO_2_ powders

#### XRD analysis

XRD analysis was performed on a Rigaku Smartlab 3 kW diffractometer equipped with a vertical goniometer. The diffractometer had a Bragg–Brentano θ/2θ measuring geometry with filtered Cu Kα radiation (λ = 1.5418 Å, *U* = 40 kV and *I* = 30 mA). The samples were measured in the range of 10–60°. Measurement steps of 0.02° at a scan rate of 2°/min were used. A 1D strip detector (Dtex250) was used during the analysis.

#### Specific surface area

The SSA of LiCoO_2_ was determined using the BET nitrogen adsorption isotherm method. The measurements were carried out with a QUADRASORB evo instrument (QUANTACHROME Instruments, USA). Before measurements, the samples were degassed for 5 h under vacuum. LiCoO_2_ was degassed at 200 °C. The purpose of degassing is to remove impurities from the surface of the tested material. Adsorption measurements were performed with nitrogen at 77 K. For each sample, an analysis was performed at 15 measurement points in the range of 0.05–0.3 *P*/*P*_0_ (relative pressure). This is the range of the SSA study. Above this range, the trend line bends. Hence, the averaging of the measurement results is inaccurate, and the results become unreliable [60].

#### SEM analysis

The morphology of the LiCoO_2_ crystallites was determined using a scanning electron microscope Auriga CrossBeam Workstation (Carl Zeiss). Cross sections of the fabricated laminates were also observed using this microscope. The SEM images show the morphology of the LiCoO_2_ obtained at different synthesis temperatures. Before analysis, the samples were sputtered with graphite to improve the electrical contact. All samples were observed at 1 kV.

#### EPR analysis

Electron paramagnetic resonance (EPR) experiments were performed using an X-band ELEXSYS E500 (Bruker, Germany) spectrometer. The samples were placed in a Super High Sensitivity Probehead (Bruker, Germany) cavity and in a cryostat where the temperature was determined and stabilized using an Oxford temperature controller ITC503S (Oxford Instruments, England). The concentration of paramagnetic ions was obtained using the procedure described elsewhere [61]. Spectral simulations were performed by applying the EasySpin software (version 5.2.27) [62].

#### Electrical properties

The electrical properties of the investigated LiCoO_2_ doped with Mn were studied using impedance spectroscopy. For conductivity measurements, pellets of ca. 1.5 mm thickness and 5.15 mm in diameter were prepared from the synthesized material. The powder was pressed at 20 MPa pressure for 1 min at room temperature. The round surfaces of the samples were covered with silver paste (Hans Wolbring GmbH) to form electrodes. IS measurements were performed using a Novocontrol AlphaA Broadband Dielectric/Impedance Spectrometer (Novocontrol GmbH) at room temperature. Measurements were carried out in the frequency range from 1 Hz to 1 MHz with an oscillation voltage of 1 V.

## Results and Discussion

### XRD analysis

**Figure 2:**
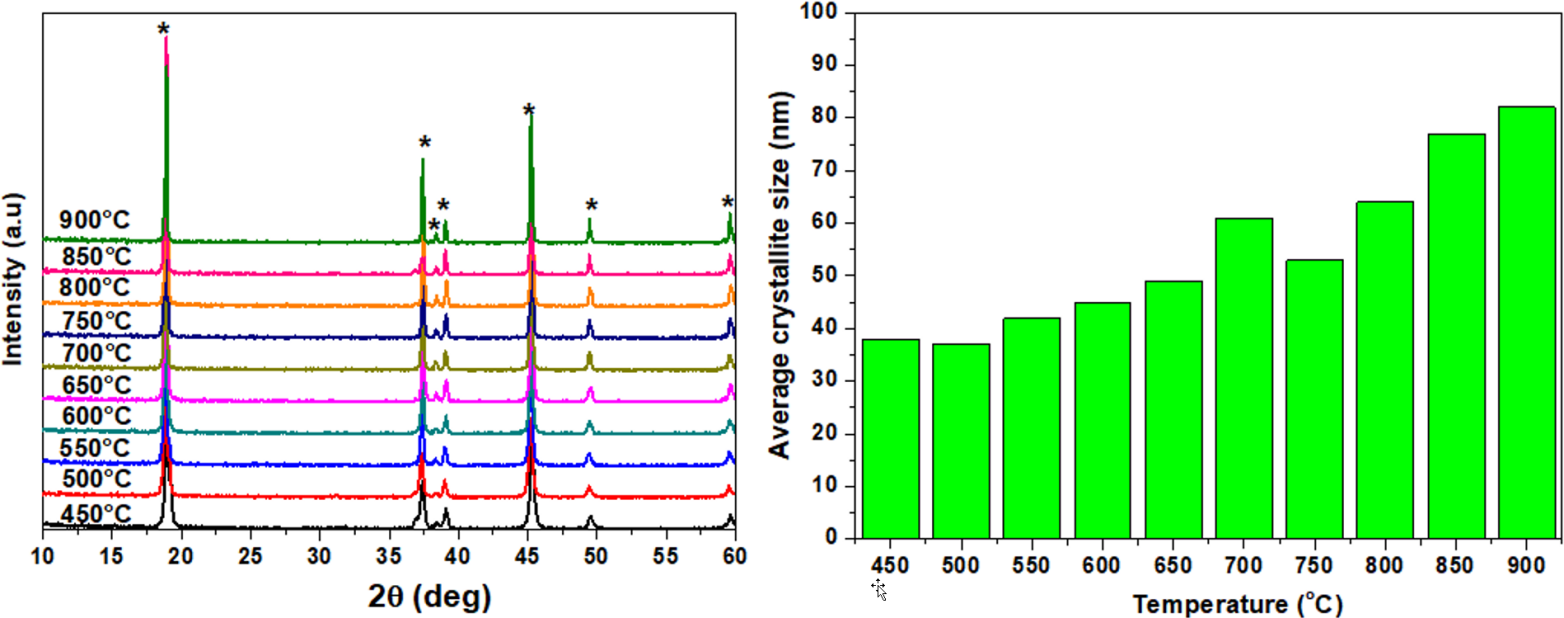
Left: XRD patterns of LiCoO_2_ heated at different temperatures (450–900 °C); right: average crystallite size as function of the temperature.

XRD analysis shows that all synthesized LiCoO_2_ powders synthesized between 450 to 900 °C (Figure 2) contain only one single phase. All powders have the hexagonal 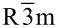 structure (ICDD PDF card – 50-0653).

The diffractograms of LiCoO_2_ powders heated at different temperatures from 450 to 900 °C with steps of 50 °C feature seven characteristic peaks at 2θ angles of 15.2°, 37.3°, 38.9°, 39.0°, 45.4°, 49.7°, and 60.0° for Cu Kα radiation (λ = 1.542 Å), corresponding to the hexagonal (rhombohedral) 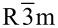 crystal planes (003), (101), (006), (012), (104), (015), and (107), respectively. Phase impurities were not detected by XRD for any of the measured samples. The average size of the crystallites depends on the heating temperature used for the combustion synthesis of powders and lies between 37 and 90 nm (as calculated from the Scherrer formula taking into account instrumental line broadening).

Average crystallite sizes, lattice parameters, and cell volumes for all nanocrystalline powders of lithium cobalt oxide are listed below in Table 1. The unit cell parameters and the unit cell volumes of LiCoO_2_ powders calculated from the XRD data are consistent with the standard values (*a*_0_ = 2.81498 Å, *c*_0_ = 14.0493 Å, *V*_0_ = 96.41 Å^3^) of the ICDD PDF card. All the lattice constants are similar to “ideal” patterns (in the PDF4+2021 ICDD database, the lattice constants for a fixed stoichiometric dispersion pattern are *a* = 2.792–2.856 Å, *c* = 14.033–14.289 Å) and are within the range given in the literature on the layered structure. The lattice constants depend on the fraction of vacancies and the mixing of Li/Co positions. There were no significant changes of the lattice constants *a* and *c* as functions of the temperature and size of the crystallites.

### Specific surface area measurements

**Figure 3:**
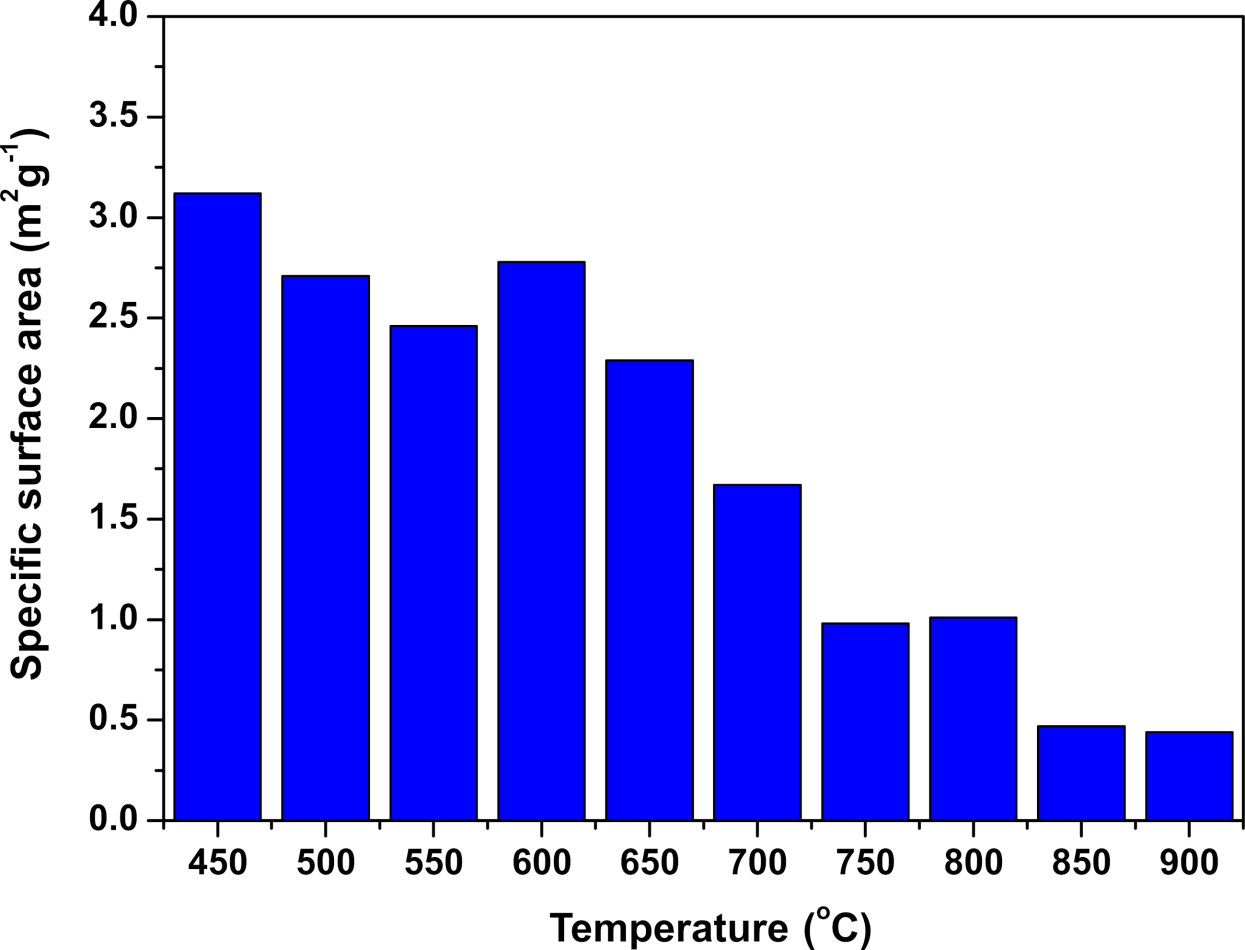
Specific surface area of LCO powders as function of the temperature during combustion solution synthesis.

Table 1 shows the results of the SSA measurements determined by the BET adsorption isotherm method. The SSA value decreased with increasing temperature, and, thus, the size of the crystallites increased, as expected. The highest value was obtained for the sample heated at 450 °C, while the lowest was obtained for the sample heated at 900 °C. The decrease in the SSA value was approximately 86%. Figure 3 shows the SSA measurements of LiCoO_2_ powders as a function of the synthesis temperature.

**Table 1:** Lattice parameter, cell volume, average crystallite size (XRD), and specific surface area of LiCoO_2_ powders.

Heating temperature [°C]	Average crystallite size [nm]	Lattice parameter *a* [Å]	Lattice parameter *c* [Å]	Volume cell V [Å^3^]	SSA (BET) [m^2^/g]

450	38	2.814	14.040	96.283	3.12
500	37	2.813	14.029	96.134	2.71
550	42	2.814	14.049	96.331	2.46
600	45	2.813	14.044	96.238	2.78
650	49	2.813	14.043	96.246	2.29
700	61	2.815	14.067	96.557	1.67
750	53	2.814	14.060	96.402	0.98
800	64	2.814	14.038	96.275	1.01
850	77	2.814	14.044	96.307	0.47
900	82	2.816	14.058	96.510	0.44

### SEM analysis

**Figure 4:**
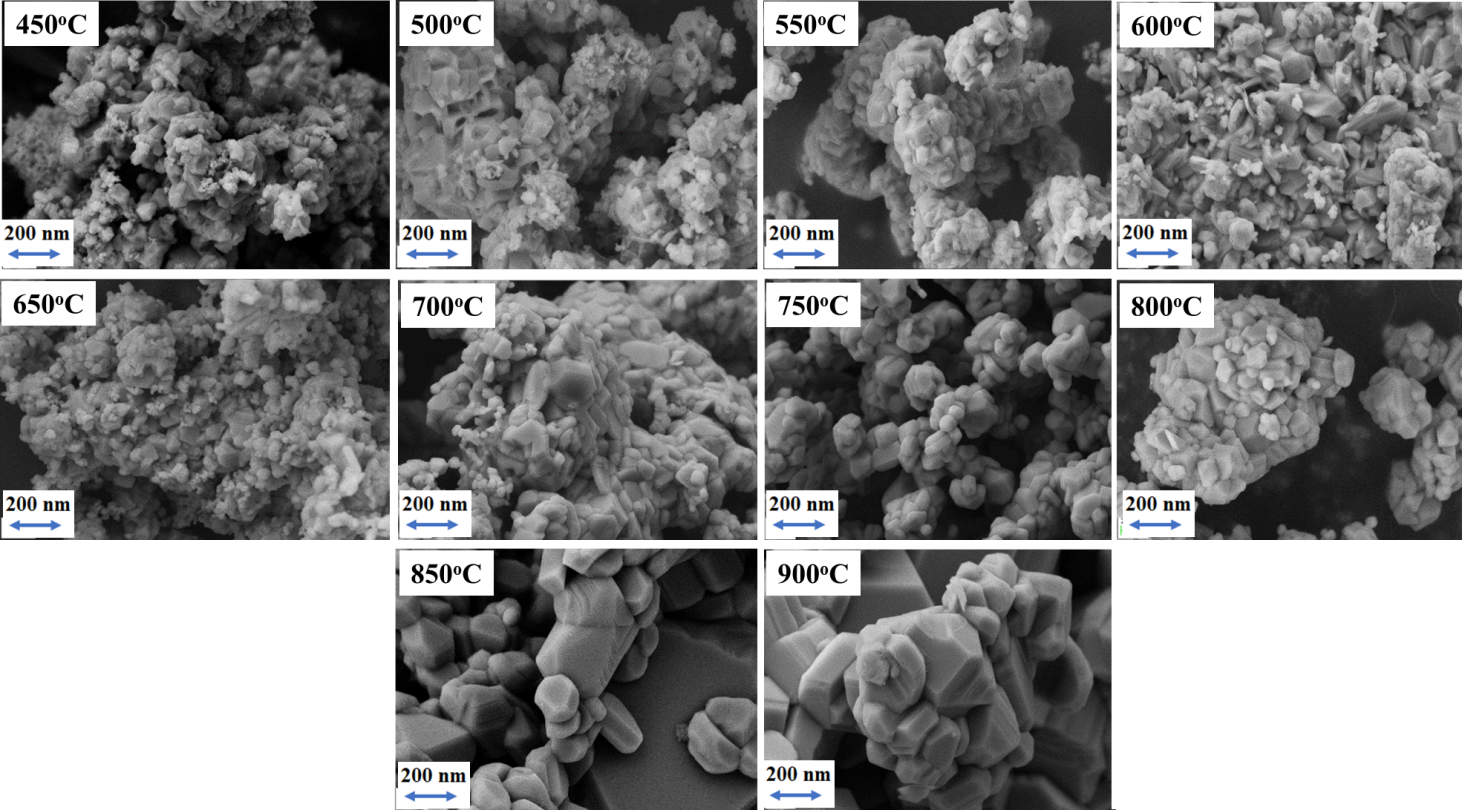
SEM images of LiCoO_2_ heated at different temperatures (450–900 °C) at 25,000× magnification.

Figure 4 shows typical SEM images of all obtained powders. Images were acquired at a magnification of 25,000×. The images show the change in morphology of LiCoO_2_ samples synthesized at different temperatures. It can be seen that, as the temperature increases, the morphology changes. Increased temperature results in significant grain growth. This can be closely correlated with the results of specific grain size and surface area measurements (Figure 3). A gradual change in the shape and the size of the crystallites is also evident. The higher the temperature, the more regular and less agglomerated the powders.

### EPR studies

**Figure 5:**
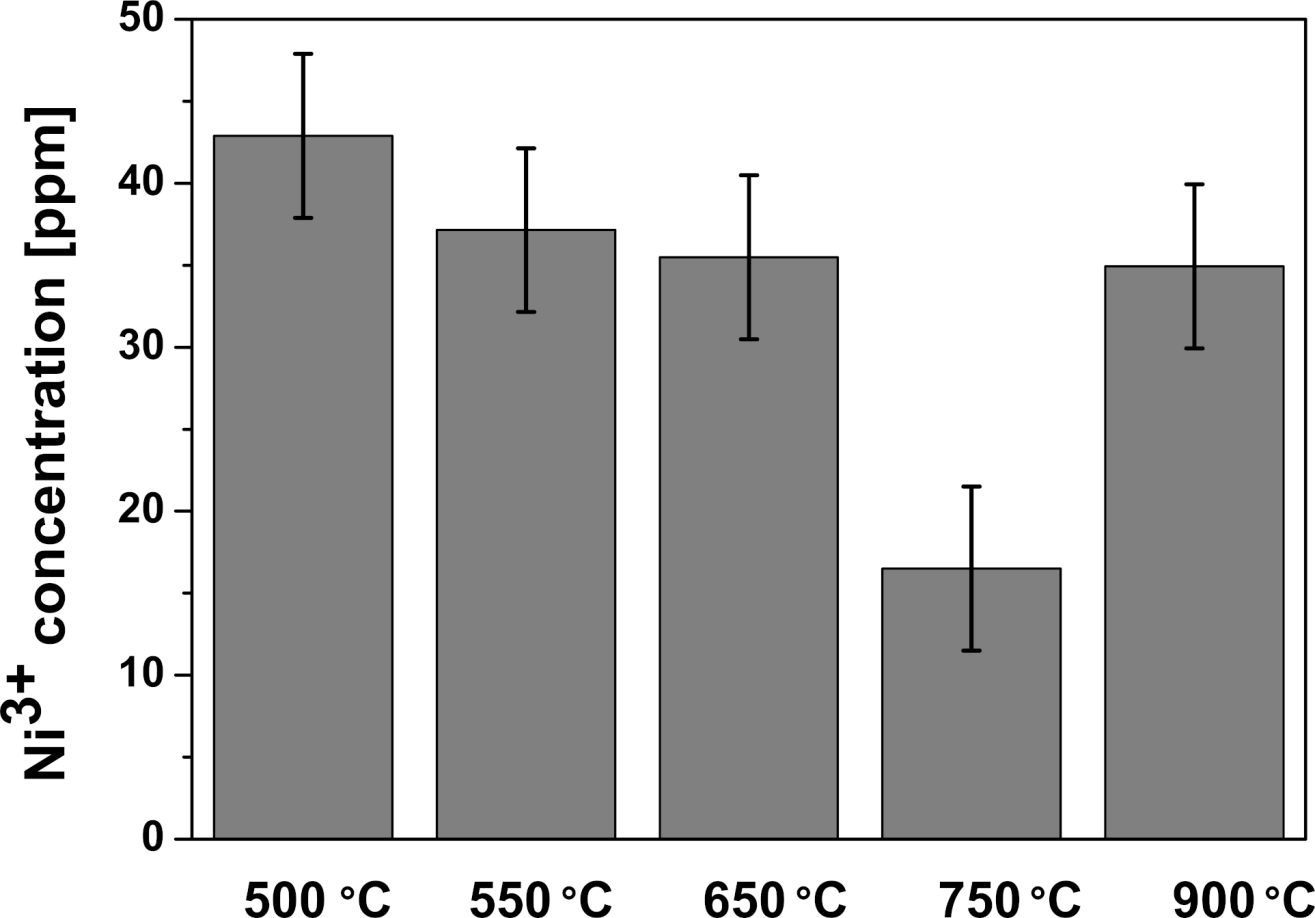
Ni^3+^ impurity concentration depending on the synthesis temperature.

EPR spectra of LCO powders consist, at room temperature, of a single symmetric line with an isotropic *g*-factor of 2.142, which indicates isolated Ni^3+^ ions in the crystal structure [63]. The low concentration of Ni^3+^ impurities (35–43 ppm) presented in Figure 5 is almost independent of the synthesis temperature. The exception is the sample heated at 750 °C with the lowest impurity concentration of the order of 17 ppm.

**Figure 6:**
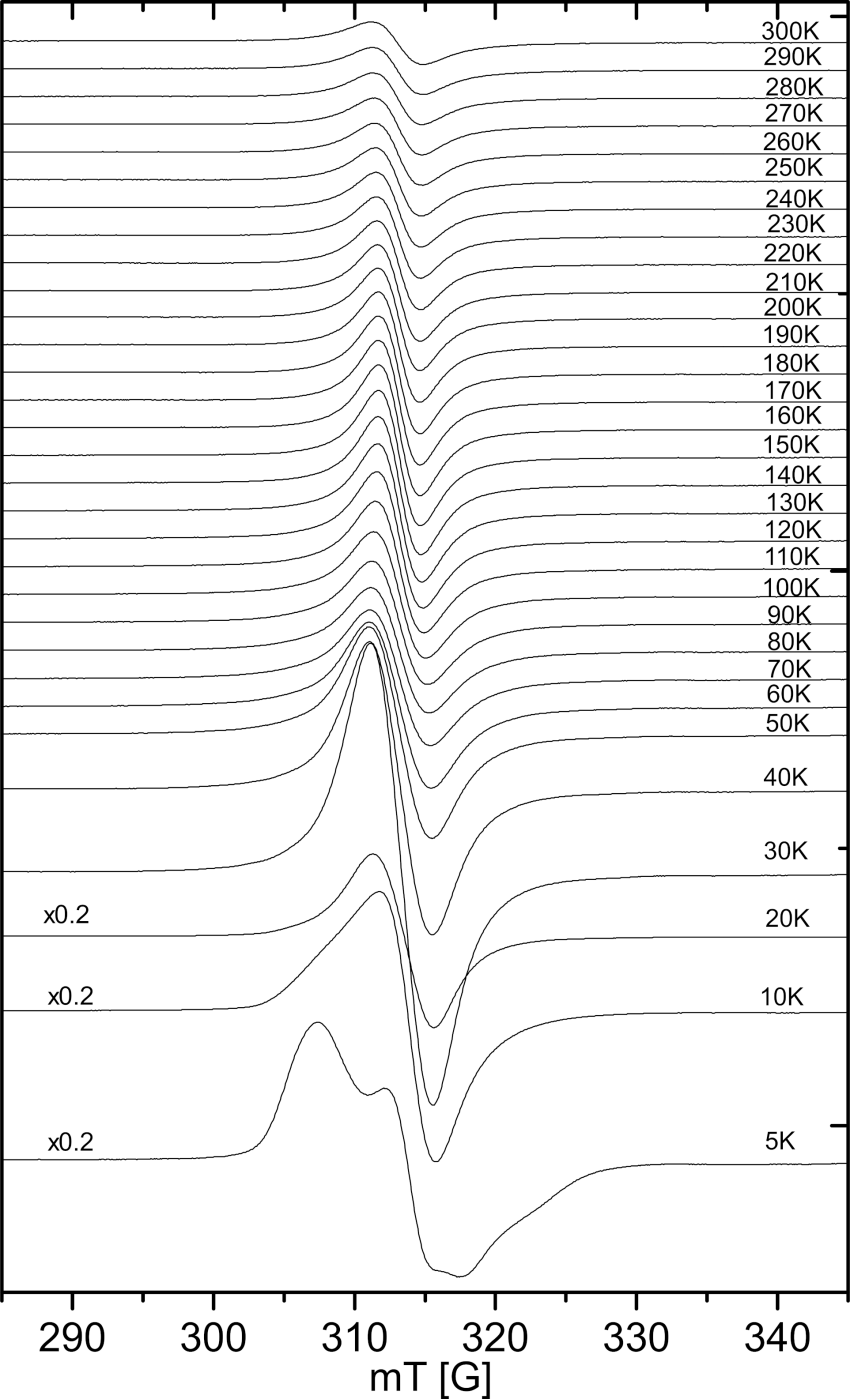
EPR spectra recorded in the temperature range of 5–300 K for LCO synthesized at 900 °C.

The spectral changes as function of the EPR measurement temperature for the sample synthesized at 900 °C are shown in Figure 6. Above about 20 K, a single isotropic line is visible, which represents the average of the spectroscopic splitting tensor due to the dynamic Jahn–Teller effect. Below about 20 K the dynamic effect becomes static, and the spectra show a clear anisotropy due to static Jahn–Teller distortion.

**Figure 7:**
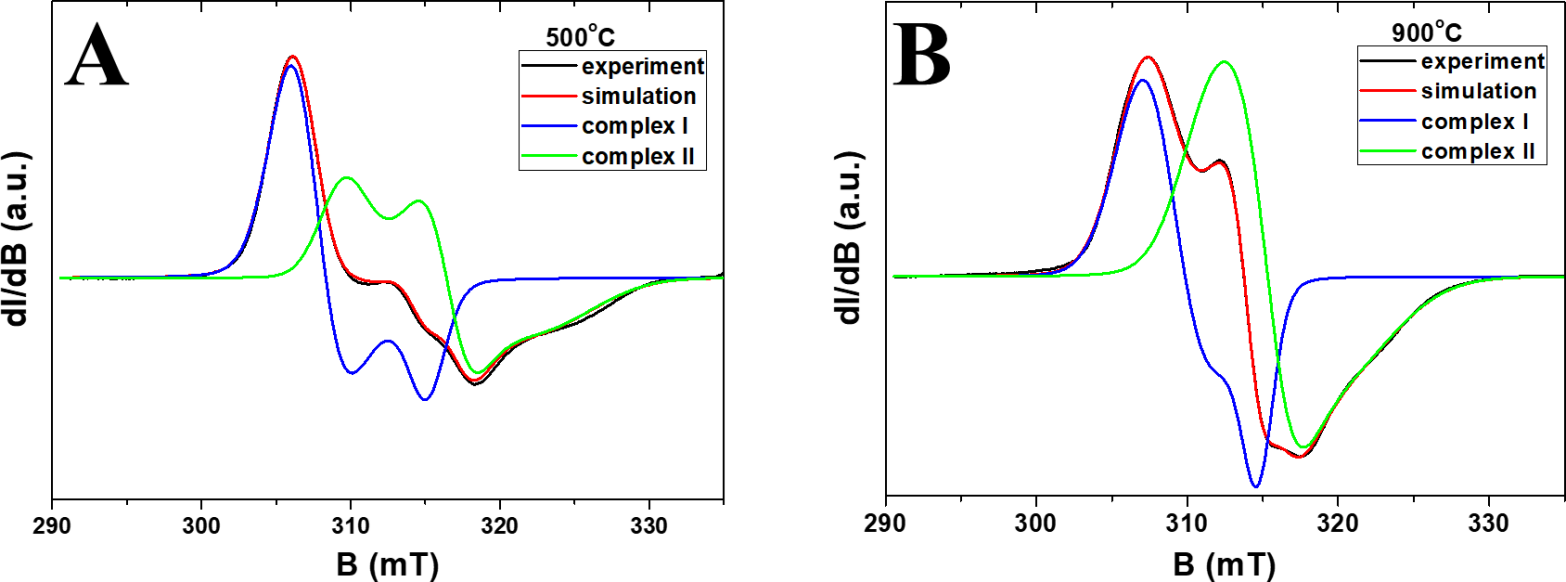
Experimental and simulated spectra for samples prepared at 500 °C (a) and 900 °C (b) and recorded at 5 K.

**Figure 8:**
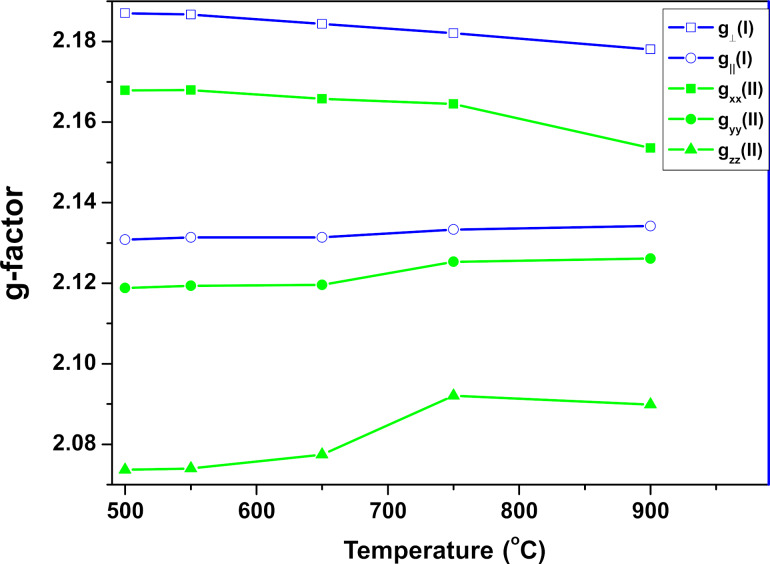
Components of the *g*-tensor for axial (complex I) and non-axial (complex II) Ni^3+^ ions.

Experimental spectra obtained at 5 K (Figure 7) show that the EPR spectra for each sample consist of two different nickel complexes. From the simulation of the experimental spectra, we obtained the parameters of the spectroscopic coefficients of the *g*-tensor for the two Ni^3+^ ions, which are presented in Figure 8. The spin-Hamiltonian parameters indicate that complex Ni(I) has axial symmetry and complex Ni(II) is non-axial. The values of the components of the *g*-tensor (Figure 8) show that the distortion of the coordination environment of Ni^3+^ ions (both axial and non-axial) decreases with the temperature at which the samples were prepared.

**Figure 9:**
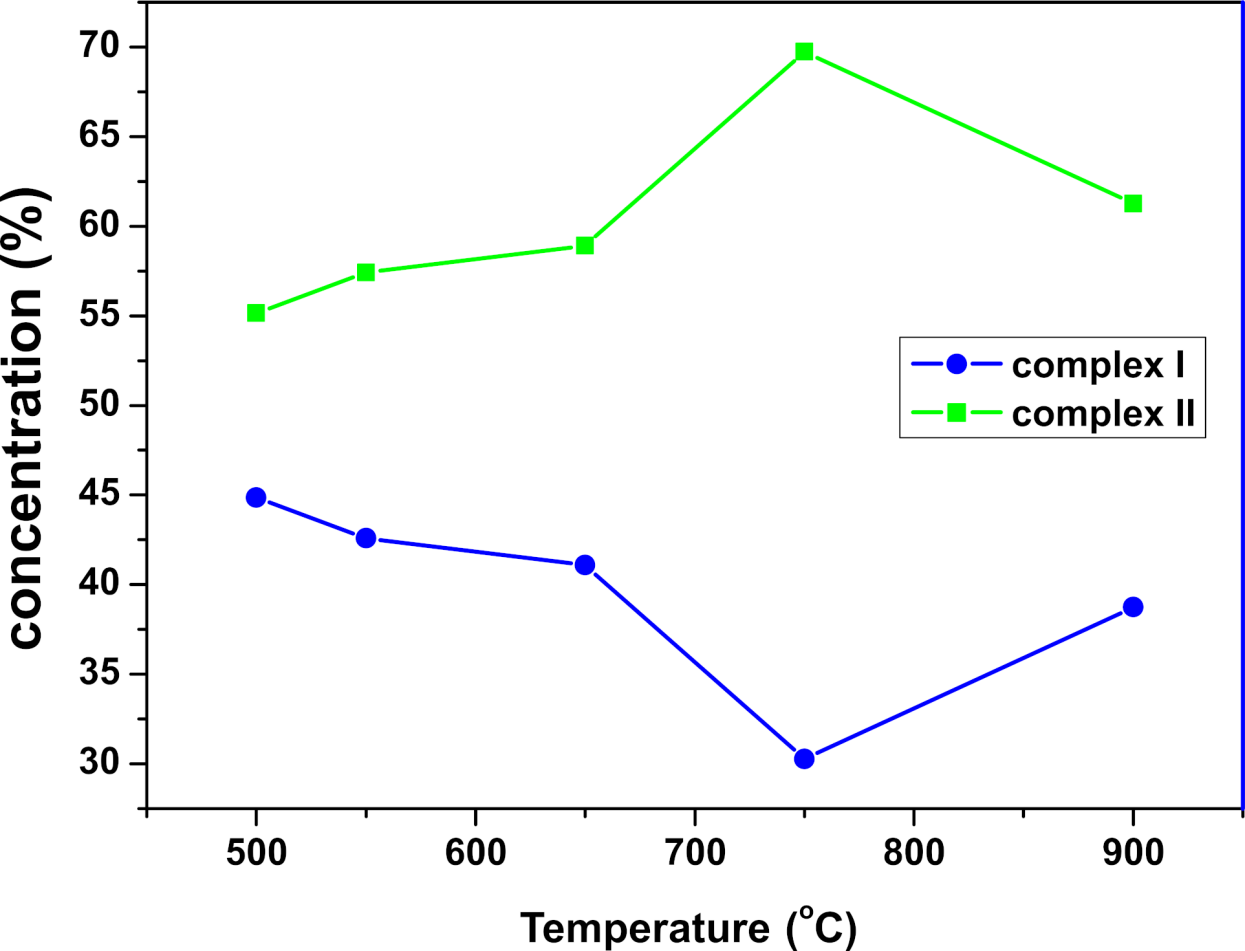
Percentage contribution of Ni^3+^(I) and Ni^3+^(II) depending on the synthesis temperature obtained from the intensity of simulated EPR spectra.

There may be two reasons for the existence of two types of nickel complexes in the spectrum. The first reason may be the expected differences in the *g*-tensor values from nickel complexes on the surface and inside the crystallites. However, as shown in Figure 9 the mutual changes in the concentration of Ni^3+^(I) and Ni^3+^(II) complexes are too small in relation to the changes in surface area measured by X-ray experiments. Therefore, the assumption that the two different nickel complexes are derived from the crystallites and the surface can probably be rejected.

The second reason why two complexes are visible in the EPR spectra is the assumption that two LCO phases are formed during the synthesis: a low-temperature cubic phase (LT-LCO) and a high-temperature trigonal phase (HT-LCO) [64–65]. In this case, Ni^3+^ (I) in the axial symmetry could be attributed to the cubic phase, and the non-axial complex Ni^3+^ (II) would be characteristic of the trigonal phase. If the nickel admixture is equally well incorporated into both phases, the percentage value of the concentration of individual nickel complexes would also be the percentage value of the individual phases in the tested samples. However, XRD studies clearly indicate that the material has only one phase.

### Electrical measurements

**Figure 10:**
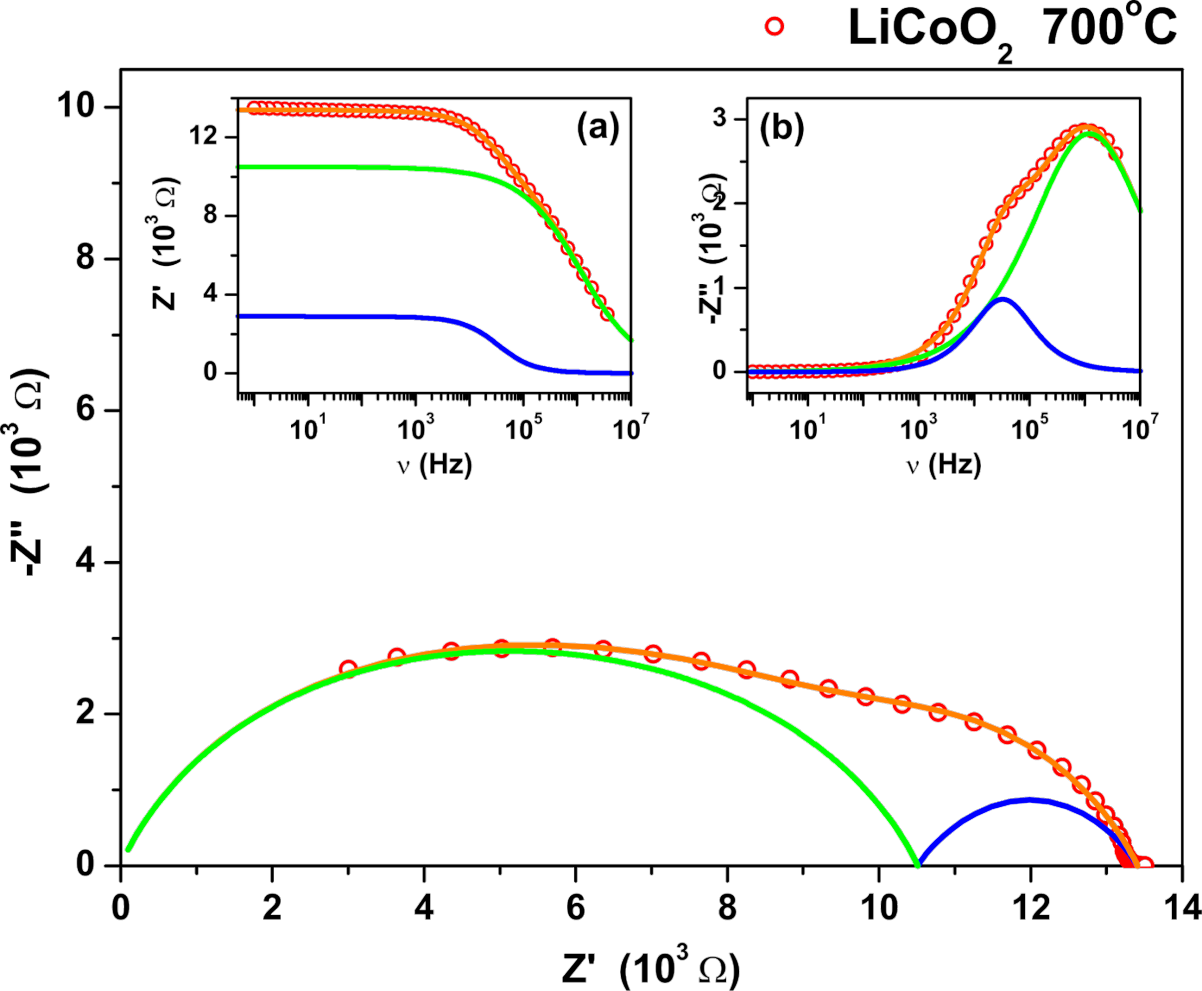
Nyquist plot of LiCoO_2_ annealed at *T* = 700 °C. The insert shows frequency dependences of the real part of impedance *Z*′ and the imaginary part *Z*″ of the studied material. The points represent the measured data, the orange line shows the calculated total impedance of the sample, the blue line represents fitted data for the grain boundaries, and the green line shows fit data for the grain interior (crystalline part of the sample).

The electric properties of the investigated LiCoO_2_ polycrystalline samples were studied using impedance spectroscopy. The measurements were performed for seven samples annealed at selected temperatures, that is, 450, 500, 550, 650, 700, 750, and 900 °C. The typical impedance response of the studied materials is presented in Figure 10. It shows the Nyquist dependence *Z*″(*Z*′) (where *Z*′ denotes the real part and *Z*″ imaginary part of the complex impedance Z*) of the LiCoO_2_ sample annealed at 700 °C.

The measured complex Z* response can be described by two parallel RC circuits connected in series:


[1] $$Z^{*}\left(\omega \right)=\frac{R_{1}}{1+{\left(i\omega R_{1}C_{1}\right)}^{1-\alpha_{1}}}+\frac{R_{2}-R_{1}}{1+{\left(i\omega \left(R_{2}-R_{1}\right)C_{2}\right)}^{1-\alpha_{2}}},$$

where *R*_1_ denotes the resistance of the crystalline part, and *R*_2_ denotes the sum of resistance of both crystalline part and grain boundaries. *C*_1_ and *C*_2_ denote the electric capacities of the two circuits, α_1_ and α_2_ are fit parameters, ω = 2π*f* is the angular frequency of the measuring field *E*-field. The value *α* describes the flattening of the semicircle caused by the distribution of the relaxation time constants. Ideally, when only one time constant describes relaxation processes in the material (Debye-type response), the fit parameter α is close to 0 and there is no flattening of the semicircle. The distribution of the time constants is related to the microstructure of the studied material as the sample is constructed from pressed powder material of different sizes. A response consisting of two semicircles is often observed in polycrystalline samples of ion conductors and ceramics [66–67]. It is related to two different contributions to total conductivity, that is, the conductivity of the bulk material (high-frequency contribution, see insert in Figure 10) and the conductivity of the grain boundaries (low-frequency contribution. The calculated value of *R* was used to determine the DC conductivity of the bulk material σ using the known sample geometry:


[2] $$\sigma_{\text{dc}}=\frac{1}{R}\frac{d}{S},$$

where *d* denotes the thickness and *S* is the surface area of the plane-parallel sample. Table 2 presents a comparison of the DC conductivity of the LiCoO_2_ samples. A comparison of the influence of the annealing temperature on the electrical conductivity shows some very interesting behavior. With increasing temperature of annealing, the value of DC conductivity increases with a maximum temperature of 500 °C. For this temperature, the DC conductivity reaches a value of 5.01 × 10^−2^ S·m^−1^. However, an additional increase in the annealing temperature causes a steady decrease in DC conductivity. For the sample of LiCoO_2_ material annealed at 900 °C, the value of DC conductivity is 9.3 × 10^−4^ S·m^−1^. The obtained DC conductivity values are similar to those observed in the literature [68].

**Table 2:** DC conductivity of the LiCoO_2_ samples measured at 293 K. The values present only the total conductivity of the sample.

Synthesis temperature [°C]	σ_dc_ [10^−3^ S m^−1^]

450	7.18
500	50.10
550	11.41
650	9.36
700	7.95
750	3.04
900	0.93

## Conclusion

Single-phase LiCoO_2_ powders were prepared using a new combustion synthesis method. The influence of synthesis temperature on structure, morphology, and electrical parameters was analyzed. The XRD results revealed that, at the lowest temperature (450 °C), LCO powder was obtained. Furthermore, the average crystallite and grain sizes increased as the heating temperature increased from 450 to 900 °C. A gradual change in the shape of the crystallites is also evident. At higher temperatures, more regular and less agglomerated powders were obtained. The specific surface area of the lithium cobalt oxide powders varies as a function of the heating temperature. It decreases with increasing temperature as the size of the crystallites increases. Electrical studies showed that conductivity strongly depends on the synthesis temperature. The samples obtained at the highest temperature had lower (more than one magnitude) conductivity. Strong thermal hysteresis is observed in all samples. However, during cooling, the samples heated to 750 and 900 °C had much higher conductivity. The EPR studies suggest the existence of two phases for LCO, and further research on this issue will be the subject of another paper. However, XRD studies clearly indicate that the material is single-phase.
